# Immunogenicity and Breakthrough Outcomes of mRNA Booster Strategies Among Healthcare Workers During the BA.1/BA.2 Omicron Surge

**DOI:** 10.3390/microorganisms13102362

**Published:** 2025-10-14

**Authors:** Song Mi Moon, Jung Nam An, Jae Hyun Kwon, Sung Gyun Kim, Han Wool Kim

**Affiliations:** 1 Department of Internal Medicine, Hallym University Sacred Heart Hospital, Hallym University College of Medicine, Anyang 14068, Republic of Korea; 2 Department of Surgery, Hallym University Sacred Heart Hospital, Hallym University College of Medicine, Anyang 14068, Republic of Korea; 3 Department of Pediatrics, Hallym University Sacred Heart Hospital, Hallym University College of Medicine, Anyang 14068, Republic of Korea

**Keywords:** COVID-19, omicron variant, healthcare workers, heterologous booster, breakthrough infection

## Abstract

Throughout the 2019 coronavirus disease pandemic, various vaccine regimens were implemented. Real-world data comparing their effectiveness during the BA.1/BA.2 Omicron wave remain limited. We prospectively enrolled healthcare workers who had completed two doses of mRNA or ChAdOx1 (A) vaccine and received an mRNA vaccine booster (BNT162b2 (P) or mRNA-1273 (M)). Neutralizing antibody levels were measured 6 months after the primary vaccinations and 1 month post-booster vaccination using a surrogate virus neutralization assay. Breakthrough infections were identified through institutional surveillance and the national reporting system. Among 318 participants (P-P-P: 71; A-A-P: 205; A-P-P: 19; M-M-M: 23), pre-booster neutralizing activity was lowest in the ChAdOx1-primed groups. One month post-booster vaccination, the neutralizing activity exceeded 97% across all regimens. The cumulative incidence of breakthrough infection varied significantly from 43.7% (P-P-P) to 84.2% (A-P-P). In adjusted Cox models, A-P-P showed the highest infection risk (HR 2.99, 95% CI 1.65–5.42). In summary, mRNA boosters restored neutralizing activity, but during the early BA.1/BA.2 Omicron wave they were less effective in preventing infections regardless of disease severity. Therefore, antibody titers alone are insufficient for evaluating protection, underscoring the need for continuous monitoring to support timely policy decisions during epidemic surges.

## 1. Introduction

During the 2019 coronavirus disease (COVID-19) pandemic, a pivotal challenge arose with the identification of the Omicron variant in late 2021 [1]. Omicron and its sublineages rapidly replaced Delta as the dominant circulating strain. During our study period in Republic of Korea, BA.1 and BA.2 were predominant, followed later by BA.4/5 and XBB.1.5 [2]. These variants carry more than 30 spike protein mutations. Their high transmissibility and immune escape capability led to widespread breakthrough infections, even among individuals who had completed the primary vaccination series [3,4]. Although booster immunization has been shown to restore neutralizing antibody responses against Omicron, its real-world effectiveness in preventing infection remains suboptimal [5,6]. Neutralizing titers alone may not fully predict protection, as immune responses are also shaped by the nature of priming exposure. This phenomenon, referred to as immune imprinting, implies that adenoviral vector and mRNA vaccines can induce distinct recall responses upon boosting [7]. Beyond antibody quantity, differences in avidity, epitope specificity, and memory B and T cell formation may contribute to a potential disconnect between antibody titers and clinical outcomes [7,8,9].

In Republic of Korea, the COVID-19 vaccination program began in early 2021, with a phased rollout strategy based on occupational risk and age [10]. Healthcare workers (HCWs) received diverse regimens according to supply and policy shifts: some were primed with adenovirus-vectored ChAdOx1, while others received mRNA vaccines (BNT162b2 or mRNA-1273). This created a natural setting for comparing homologous and heterologous booster strategies within a relatively homogeneous occupational group [10,11].

Heterologous prime-boost strategies have been proposed for enhancing humoral and cellular immunity by stimulating various immunological pathways. Several immunogenicity studies have demonstrated that adenovirus-vectored priming, followed by mRNA boosting, can induce stronger or comparable neutralizing antibody responses to homologous regimens [12,13]. A recent systematic review suggested no consistent advantage of homologous over heterologous strategies in terms of breakthrough infection or severe disease, especially during the Omicron wave [14]. This distinction may reflect the different immunological correlates of protection: neutralizing antibodies are primarily associated with preventing infection, whereas T cell responses play a greater role in limiting severe outcomes [7,15].

However, most existing evidence is derived from controlled trials or meta-analyses across heterogeneous populations. Real-world cohort data for evaluating immune responses and clinical outcomes in occupationally homogeneous, vaccine-diverse settings are lacking. We hypothesized that while heterologous mRNA boosters could restore neutralizing antibody levels after adenovirus-vector priming, qualitative immune differences between vaccine platforms might still result in divergent risks of breakthrough infection. To address this gap, our goal was to assess whether heterologous mRNA vaccine boosters restored neutralizing antibody levels, and to determine whether neutralizing titers adequately predicted protection against breakthrough infection. We showed that heterologous mRNA vaccine boosters restored neutralizing antibody levels; however, neutralizing titers alone may be an insufficient predictor of protection.

## 2. Materials and Methods

### 2.1. Study Design

**Figure 1:**
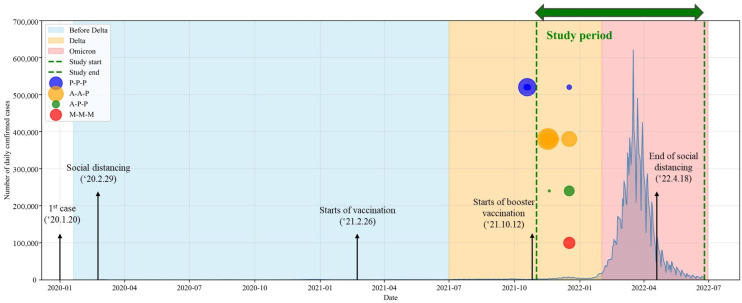
National daily confirmed 2019 coronavirus disease (COVID-19) cases in Republic of Korea with major variant periods and timing of booster vaccination among study participants. Daily case numbers were obtained from the KDCA (https://dportal.kdca.go.kr/pot/cv/trend/dmstc/selectMntrgSttus.do accessed on 20 August 2025). Shaded areas indicate Delta- and Omicron-dominant periods. Vertical dashed green lines represent the study observation period (1 November 2021–25 June 2022). Colored dots represent booster vaccination dates of study participants stratified by booster regimen (P-P-P, A-A-P, A-P-P, M-M-M), with dot size proportional to the number of participants vaccinated on that date.

We conducted a prospective study of adult HCWs working at Hallym University Sacred Heart Hospital, a tertiary referral center with over 800 beds in Republic of Korea, from November 2021 to June 2022. Enrollment was open to all HCWs identified by the infection control office as due for a third (booster) dose 6 months after completion of their primary vaccination series. The primary vaccine regimen varied and included two mRNA vaccines and one vector vaccine (ChAdOx1, Vaxzervia, AstraZeneca AB, Södertälje, Sweden). They received a booster dose of one of two mRNA vaccines (BNT162b2, Comirnaty, Pfizer Inc., New York, NY, USA, P; or mRNA-1273, Spikevax, Moderna, Princeton, NJ, USA, M). Four types of regimens were used (P-P-P, M-M-M, A-A-P, and A-P-P). The distribution of vaccine regimens reflected national allocation policies and occupational risk rather than personal choice [10,11]. During the initial rollout in Republic of Korea, vaccine procurement and approval were staggered, resulting in age- and occupation-based allocation. Older individuals and high-risk staff were prioritized for adenovirus-vectored vaccines (ChAdOx1 or single-dose of Ad26.COV2.S [Janssen Pharmaceuticals Companies of Johnson & Johnson, Titusville, NJ, USA]), while younger healthcare workers and the general population more frequently received mRNA vaccines (BNT162b2 or mRNA-1273). At our institution, these policies directly influenced vaccine assignment across occupational groups, leading to heterogeneous primary regimens in the workforce. For epidemiological data, national daily confirmed COVID-19 cases were obtained from the Korea Disease Control and Prevention Agency (KDCA; https://dportal.kdca.go.kr/pot/cv/trend/dmstc/selectMntrgSttus.do; accessed on 20 August 2025), and the study period was overlaid on the epidemic curve (Figure 1). This was because breakthrough infections were likely to have been strongly influenced by the magnitude of community outbreaks.

### 2.2. Demographics and Serum Samples

We collected demographic data, including sex, age, and occupational information, classified according to whether they were in clinical roles (such as physicians, nurses, therapists) or non-clinical administrative/support roles (such as administrative staff, clerical workers, and support services). Sera from the HCWs were collected 6 months after the second dose of the severe acute respiratory syndrome-2 (SARS-CoV-2) vaccine and again 1 month after the third dose.

### 2.3. Immunogenicity Assessment

Serum samples were collected to assess humoral responses at two time points: immediately before the third (booster) dose (V1) and 1 month after the booster dose (V2). To assess the potential neutralizing capacity of the HCW sera, the cPass™ SARS-CoV-2 Neutralization Antibody Detection Kit (GenScript Biotech, Mainz, Germany) was used. This assay employs the spike receptor binding domain (RBD) of the ancestral Wuhan strain to measure inhibition of RBD- angiotensin-converting enzyme 2 interaction [16]. The samples were diluted tenfold according to the manufacturer’s instructions. Sample values below the threshold of 30% were considered negative, and values at or above the cut-off indicated the presence of SARS-CoV-2 neutralizing antibodies. At this cutoff, the negative and positive percentage agreements with the conventional plaque reduction neutralization test (PRNT) 50 and PRNT90 assays were approximately 100%. The manufacturer reported the sensitivity and specificity of the assay to be 93.8% and 99.4%, respectively. Inhibition (%) was calculated as (1 − OD value of sample/OD value of negative control) × 100. Seropositivity was defined as inhibition ≥30%. ‘High responder’ was defined a priori as inhibition ≥90%.

### 2.4. Breakthrough Infection Rate

The incidence of COVID-19 among participants was evaluated to assess vaccine effectiveness. Breakthrough infections were defined as laboratory-confirmed SARS-CoV-2 cases detected after booster vaccination. According to national policy during the study period, all symptomatic individuals and close contacts of confirmed cases were required to undergo PCR testing, while asymptomatic routine follow-up screening was not performed for vaccinated HCWs. At our institution, the infection control office actively monitored all in-hospital confirmed cases, conducted contact tracing, and required HCWs to promptly report any COVID-19 diagnosis, including those identified in the community.

To describe the temporal distribution of breakthrough infections, scatter plots were constructed by days since the booster. Histograms and rug plots were used to display the infection events across regimens, and these were overlaid with the national epidemic curve data obtained from the KDCA. For infection-free probability analyses, only the first breakthrough infection was considered, with follow-up censored at study end. Two time origins were prespecified: (i) the individual booster date and (ii) a common start date (17 December 2021).

### 2.5. Statistical Analysis

**Figure 2:**
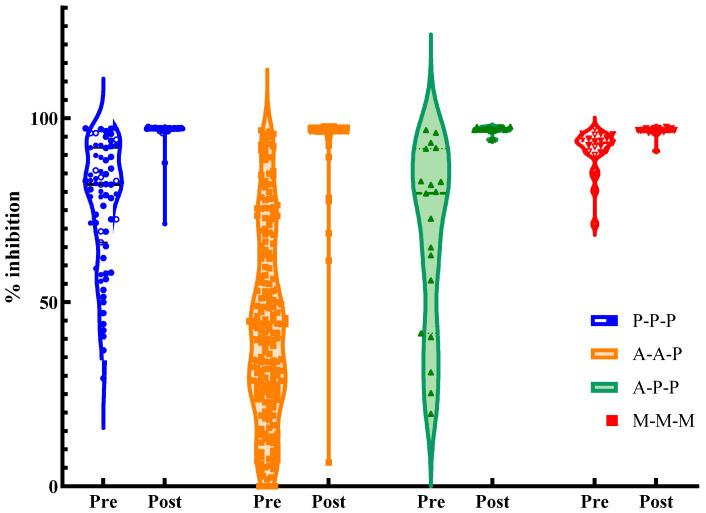
Violin plots of neutralizing antibody inhibition (%) at baseline (V1, pre-booster) and 1 month post-booster (V2) according to vaccine regimen. Each point represents an individual, and horizontal bars indicate the median and interquartile range (IQR). Vaccine regimens are denoted as follows: P-P-P = BNT162b2 (Pfizer-BioNTech) for all three doses; A-A-P = two doses of ChAdOx1 (AstraZeneca) followed by a BNT162b2 booster; A-P-P = one dose of ChAdOx1 and two subsequent doses of BNT162b2; M-M-M = mRNA-1273 (Moderna) for all three doses.

Descriptive statistics were used for demographic data. Continuous variables were expressed as mean ± standard deviation or median with interquartile range (IQR), depending on the distribution. The Kruskal–Wallis test was used to compare continuous variables among the groups, and post hoc pairwise comparisons were performed using the Mann–Whitney U test with Bonferroni correction. The chi-squared test or Fisher’s (Fisher–Freeman–Halton for r × c table) exact test was used to compare categorical variables. Pre- and post-booster inhibition values were compared within groups using the Wilcoxon signed-rank test, and the Kruskal–Wallis test was used for between-group comparisons. Multiple testing correction was applied consistently to all post hoc pairwise comparisons of continuous outcomes (e.g., antibody inhibition levels and absolute changes between regimens), whereas infection-free probability analyses and Cox regression were treated as prespecified global or model-based analyses and were not adjusted. Kaplan–Meier survival curves illustrating infection-free probability were used to compare the probability of remaining free from breakthrough infection following the booster regimen, and differences were tested using the log-rank test. Cox proportional hazards models were fitted to estimate the hazard ratios (HRs) with 95% confidence intervals (CIs) after adjusting for age and sex. The proportional hazards assumption was evaluated using Schoenfeld residuals (Grambsch–Therneau test) and log(−log) infection-free probability plots (Figures S1–S3). Figures were generated using Python 3.10 (matplotlib, seaborn, and lifelines). Cox proportional hazards models estimated HRs with 95% CIs, using P-P-P as the reference category and adjusting for age and sex. In a sensitivity model, occupation (clinical vs. non-clinical) was additionally included. Immunogenicity plots (Figure 2) were generated using GraphPad Prism version 10.1.0 (GraphPad, San Diego, CA, USA). Differences were considered statistically significant at two-tailed *p*-values < 0.05.

## 3. Results

### 3.1. Baseline Characteristics

Of 494 healthcare workers (HCWs) eligible for a booster dose 6 months after their primary vaccination, 318 (64.4%) consented to participate in this prospective cohort. The eligible population included P-P-P (*n* = 99), A-A-P (*n* = 255), A-P-P (*n* = 63), and M-M-M (*n* = 77). Among enrolled participants, 71 received P-P-P, 205 received A-A-P, 19 received A-P-P, and 23 received M-M-M. Baseline characteristics of the enrolled cohort are summarized in Table 1, with a median age of 32 years and 74.8% female. Age distribution differed significantly among the regimens (*p* < 0.001). The highest median age was observed in the A-P-P group (41 years, IQR: 34–44 years), followed by the A-A-P group (35 years, IQR: 27–49 years). The P-P-P (28; IQR, 26–37) and M-M-M (25; IQR, 23–31) groups included younger participants. Post hoc comparisons showed that A-P-P participants were significantly older than those in P-P-P and M-M-M, while no significant differences were found between A-P-P and A-A-P or between P-P-P and M-M-M (Table 1). This imbalance most likely reflects national allocation policies during the initial rollout, wherein higher-risk older adults were prioritized for vector-based priming, and mRNA-1273 was preferentially recommended to younger adults due to adverse-event concerns [17,18].

Clinical occupations, defined as patient-facing roles, such as physicians, nurses, and therapists, accounted for 73.6% of the total population. Seven participants (2.2%) had SARS-CoV-2 infection before the booster vaccination.

The median follow-up duration also differed significantly across booster groups (*p* < 0.001). Post hoc analyses indicated longer follow-up in P-P-P than in A-A-P, A-P-P, and M-M-M (all adjusted *p* < 0.001). A-A-P differed from A-P-P and M-M-M (adjusted *p* < 0.01), whereas A-P-P and M-M-M did not differ. These findings indicate an imbalanced distribution of follow-up time (Table 1). Given these context-driven imbalances in age, occupation, and follow-up, we prespecified adjusted Cox models (age and sex; occupation in sensitivity analyses) and performed a common time-origin infection-free survival analysis aligned to the epidemic phase to mitigate confounding by calendar time and time-since-booster.

### 3.2. Immunogenicity After Booster Vaccination

Overall, booster vaccination substantially increased neutralizing antibody levels across all regimens (Table 2, Figure 2). Table 2 summarizes the immunogenicity results before and after booster vaccination. Before the booster vaccination, inhibition (%) differed significantly between groups, with post hoc analysis showing that it was significantly lower in the A-A-P group than in the P-P-P, M-M-M, and A-P-P groups. Seropositivity rates were substantially lower in the A-A-P group (64.9%) than in the other groups, exceeding 89%. The virus vector vaccine regimen showed lower antibody levels 6 months after the primary series. After 1 month, inhibition (%) increased sharply for all regimens (median values ~97), with P-P-P the highest, A-P-P the lowest, and A-A-P and M-M-M intermediates. Seropositivity rates exceeded 99% for all regimens after the booster vaccination. The absolute changes in antibody levels after booster vaccination (V2–V1) were calculated for each regimen (Table 2). The median (IQR) absolute change was 55 (34–71) in the A-A-P group, followed by the A-P-P, P-P-P, and M-M-M groups. In the M-M-M group, this smaller increment likely reflects a ceiling effect due to already high pre-booster levels. Post hoc pairwise comparisons with Bonferroni correction showed that A-A-P had significantly greater increases than A-P-P, M-M-M, and P-P-P (all *p* < 0.01), whereas A-P-P and P-P-P did not differ significantly (*p* = 1.00). The proportions of high responders (≥90% inhibition) were 98.6%, 95.7%, 93.7%, and 73.7% in the P-P-P, M-M-M, A-A-P, and A-P-P groups, respectively. In addition, no significant difference in inhibition (%) at 1 month was observed between participants who subsequently experienced breakthrough infection and those who did not. Median post-booster values were nearly identical in both groups (e.g., P-P-P 97.2% vs. 97.2%; A-A-P 96.9% vs. 97.0%), indicating that similar antibody titers did not necessarily translate into equivalent protection.

### 3.3. Breakthrough Infections

**Figure 3:**
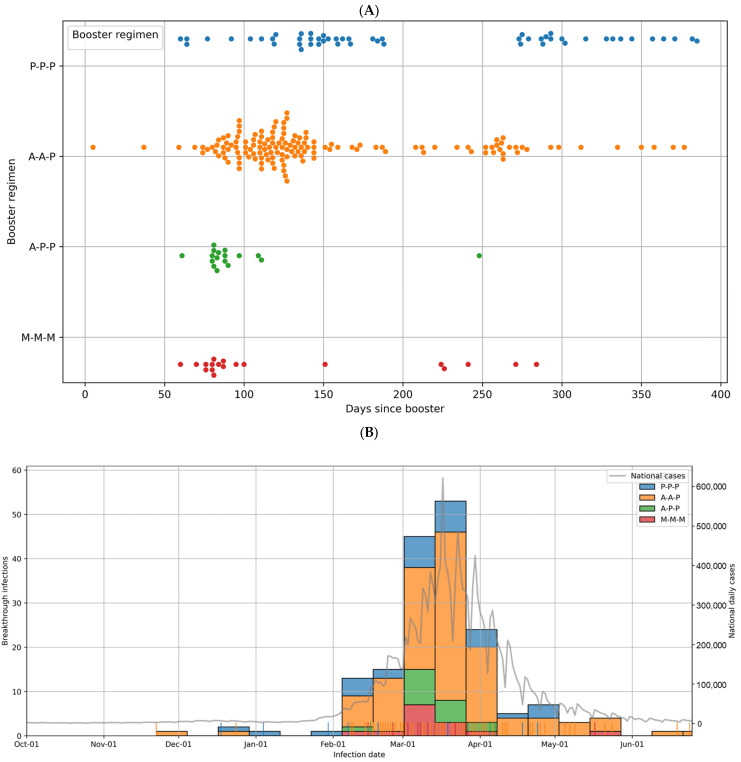
Breakthrough infection distribution by days since booster vaccination and by calendar date. (**A**) Distribution of breakthrough infections by days since booster vaccination. Histogram and rug plots showing the timing of breakthrough infections after booster vaccination across different booster regimens. Each dot represents an individual case, colored by regimen (P-P-P, A-A-P, A-P-P, M-M-M). (**B**) Distribution of breakthrough infections by calendar date during the study period. Daily distribution of breakthrough infections plotted against the national epidemic curve (gray line). Colored dots indicate individual breakthrough cases according to the booster regimen. Clustering of infections is observed during the peak of community transmission.

During the follow-up period up to 25 June 2022, 175 breakthrough infections were documented, corresponding to an overall cumulative incidence of 55.0%. Most infections occurred during the Omicron wave, which was initially dominated by BA.1/1.1 and later by BA.2/2.3 sublineages. Figure 3 shows the temporal distribution of the breakthrough infections. In Figure 3A, infections are plotted by days since the booster, showing differences in timing across regimens. In contrast, the calendar date distribution of infections is shown in relation to national epidemic trends, highlighting the clustering of breakthrough infections during the peak of community transmission in Figure 3B. The cumulative incidence of breakthrough infection varied across the regimens, ranging from 43.7% in the P-P-P group to 84.2% in the A-P-P group, with the A-A-P and M-M-M groups showing intermediate rates (56.1% and 60.9%, respectively) (Table 2). The overall differences between the regimens were statistically significant (*p* = 0.0137), and the post hoc analysis indicated a significantly higher incidence in the A-P-P group than in the A-A-P and P-P-P groups. No significant differences were observed between the other groups.

### 3.4. Breakthrough Infections: Comparison Among Booster Regimens

**Figure 4:**
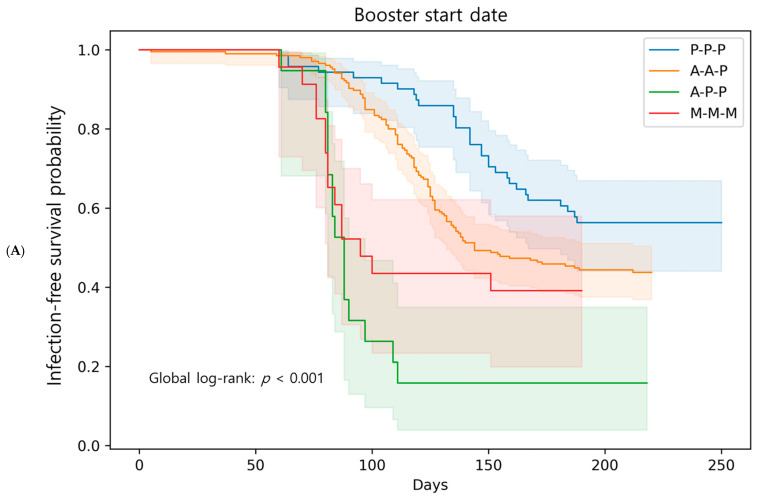

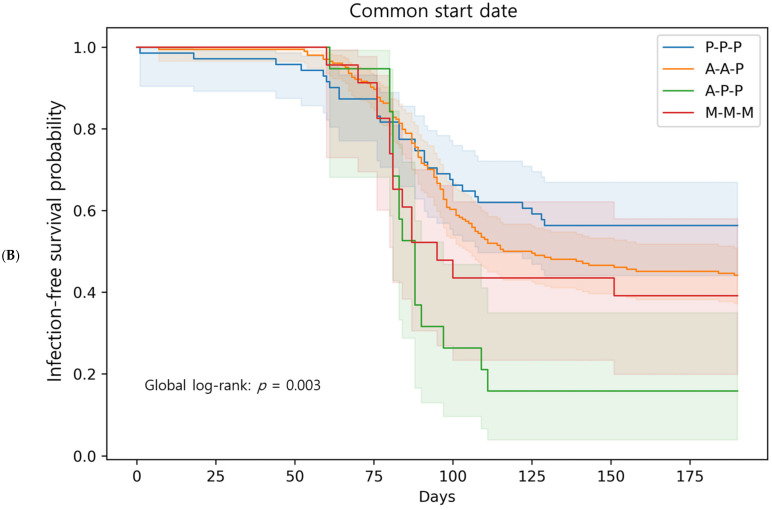
Kaplan–Meier infection-free survival curves for breakthrough infection according to booster regimen. (**A**) Infection-free survival curves with individual booster date as the start of follow-up. The P-P-P group showed the highest probability of remaining infection-free, while the A-P-P group demonstrated the steepest decline. Median follow-up durations were 249 days for P-P-P, 144 days for A-A-P, 84 days for A-P-P, and 95 days for M-M-M (Table 1) (**B**) Sensitivity analysis with 17 December 2021, when all participants had completed booster vaccination, as a common start date. In both analyses, the A-P-P group showed a significantly higher risk of breakthrough infection compared with other regimens.

The Kaplan–Meier infection-free survival curves for the booster regimen are shown in Figure 4. Using the booster date as the start of observation (Figure 4A), the P-P-P group had the highest infection-free probability without infection, whereas the A-P-P group demonstrated the steepest decline, and the A-A-P and M-M-M groups remained intermediate. Pairwise log-rank tests showed significant differences between P-P-P and A-P-P (*p* < 0.001), between P-P-P and A-A-P (*p* = 0.017), and between P-P-P and M-M-M (*p* = 0.015). In the Cox model adjusted for age and sex with P-P-P as the reference, the A-P-P regimen showed the highest risk of breakthrough infection (HR 5.82, 95% CI 2.61–12.94, *p* < 0.001). Both A-A-P (HR 1.57, 95% CI 1.09–2.27, *p* = 0.016) and M-M-M (HR 2.44, 95% CI 1.14–5.19, *p* = 0.021) also had significantly higher risks.

Because the vaccination timing differed across groups, an infection-free survival analysis was also performed using a common observation start date (17 December 2021), when all booster vaccinations had been completed (Figure 4B). In this analysis, the infection-free survival curves showed that A-P-P had the lowest infection-free survival rate, whereas P-P-P remained the most favorable. The overall separation between the groups was attenuated; however, the log-rank comparisons remained significant for A-P-P versus P-P-P (*p* < 0.001), whereas other pairwise differences were not significant. The Cox model again showed that A-P-P was significantly associated with a higher risk (HR 2.99, 95% CI 1.65–5.42, *p* = 0.0003), while A-A-P and M-M-M were not significantly different from P-P-P. In the booster-date model, Schoenfeld residual tests indicated deviations for some regimen covariates (Figure S1), whereas no violations were observed in the common-date model (Figure S2). Log(−log) infection-free survival plots appeared approximately parallel across regimens, further supporting proportionality (Figure S3).

### 3.5. Occupation-Based Analysis

When stratified into clinical and non-clinical occupations, no significant difference was observed in the infection-free survival curves (log-rank *p* = 0.43) (Figure S4). In Cox regression adjusting for age, sex, and booster vaccination regimen, occupation was not significantly associated with infection risk (HR: 0.75, 95% CI: 0.52–1.10), suggesting that infection risk was driven primarily by community transmission rather than hospital exposure.

## 4. Discussion

This prospective cohort study of 318 HCWs during the BA.1/BA.2-dominant Omicron surge in Republic of Korea demonstrated that heterologous mRNA booster vaccination effectively restored neutralizing antibody levels, regardless of the primary vaccine regimen. While participants who received adenovirus-vectored primary vaccines (ChAdOx1) had significantly lower pre-booster antibody levels (6 months after the primary series) than those who received mRNA vaccines, all groups achieved comparable neutralizing activity 1 month after the mRNA vaccine booster dose. Despite this immunological restoration, our study found high breakthrough infection rates in all the vaccination groups, with a significantly higher incidence in the A-P-P group than in the A-A-P and P-P-P groups during the BA.1/BA.2 Omicron surge.

Heterologous prime-boost vaccination strategies have emerged as a critical approach for COVID-19 immunization, particularly driven by vaccine supply constraints and the need to optimize immune responses against emerging variants [19,20]. The scientific rationale for using heterologous vaccination regimens stems from the potential to stimulate different arms of the immune system, with adenovirus vector vaccines primarily inducing cellular immunity, and mRNA vaccines generating robust humoral responses [21,22].

Multiple studies have consistently demonstrated the superior immunogenicity of heterologous booster strategies compared with homologous regimens. A randomized clinical trial in HCWs showed that mRNA vaccines as boosters following ChAdOx1 priming resulted in substantial antibody increases; BNT162b2 boosters increased anti-spike IgG by 32.2-fold, while half- and full-dose mRNA-1273 boosters increased antibodies by 47.6-fold and 63.2-fold, respectively [23].

The immunological basis for enhanced heterologous responses likely involves the activation of different immunological pathways. Adenovirus vector vaccines induce strong CD4+ and CD8+ T cell responses through their ability to present antigens via major histocompatibility complex class I pathways, whereas mRNA vaccines excel at generating high-titer neutralizing antibodies through efficient antigen presentation and innate immune activation [24,25]. Such T cell-mediated immunity, which targets conserved epitopes largely preserved against Omicron mutations [15], may provide benefits not fully reflected by serum IgG titers. Although the A-P-P regimen showed higher breakthrough infection rates, T cell responses could have contributed to attenuating the severity of these infections. Our study did not capture data on the clinical severity of breakthrough infections, so this possibility could not be directly evaluated. This complementary mechanism may explain why heterologous regimens often outperform homologous strategies in terms of breadth and magnitude of immune responses.

Moreover, immune imprinting may also influence these outcomes, as the nature of the initial priming—adenovirus-vectored versus mRNA platforms—can shape subsequent recall responses and potentially limit the breadth of neutralization against antigenically divergent Omicron sublineages [7]. Although neutralizing titers appeared comparable across regimens, qualitative features such as affinity maturation, Fc-mediated effector functions, and durability may differ by platform, thereby affecting protection beyond antibody levels alone [7,9]. Adenovirus-vectored priming has additionally been associated with trained innate immunity and enhanced T cell responses [8], yet such responses may not have been sufficient to overcome the rapid immune escape of Omicron.

These findings align with our results, where the heterologous A-A-P group showed the greatest increase in absolute antibody levels after booster vaccination, compared with smaller increases in homologous mRNA groups such as M-M-M and P-P-P. This larger absolute change in the A-A-P group likely reflects the lower baseline neutralizing antibody levels observed 6 months after the primary series with an adenoviral vector vaccine, providing a greater margin for recovery after boosting. In contrast, the P-P-P group showed a smaller absolute increase due to higher pre-booster antibody levels, yet maintained a relatively low risk of breakthrough infection during the BA.1/BA.2 Omicron surge. Taken together, the ability of the mRNA boosters used in this study to effectively restore neutralizing antibody levels regardless of the primary vaccine type demonstrates the potent immunogenicity of the mRNA vaccines. This suggests that, even when primary vaccination is performed using a regimen that induces weaker immunogenicity, an mRNA booster can effectively “rescue” the immune response, achieving comparable post-booster neutralizing activity across different primary regimens.

Despite the rescued immune status, breakthrough infections were more frequent in our cohort. The emergence of the BA.1/BA.2 Omicron variant poses major challenges to vaccine effectiveness owing to extensive mutations in the spike protein, which facilitate immune escape [26,27]. In this study, the overall cumulative breakthrough infection rate was 55%, reflecting the trend during the Omicron wave, when many boosted individuals were infected. Several large-scale studies have reported the reduced effectiveness of all vaccines against the Omicron variant. A Swedish nationwide cohort study showed that heterologous vaccination using ChAdOx1, followed by BNT162b2 or mRNA-1273, achieved 79% effectiveness against symptomatic COVID-19 infection during the delta-dominant period; however, this effectiveness dropped substantially during the Omicron wave [28]. Similarly, serological studies have consistently shown reduced neutralizing activity against the Omicron compared with the wild-type virus across all vaccine combinations [23,29].

Subsequent vaccination strategies have changed owing to issues with low efficacy against Omicron. The importance of adaptive vaccination strategies has been further demonstrated by the rapid development and deployment of variant-adapted vaccines in response to the evolving SARS-CoV-2 strains. Following the emergence of Omicron and its subvariants, regulatory agencies approved bivalent vaccines targeting both the original strain and Omicron variants. The Food and Drug Administration authorized Pfizer-BioNTech and Moderna bivalent vaccines in August 2022, specifically targeting the BA.4/BA.5 subvariants [30,31]. Subsequently, updated monovalent vaccines targeting the XBB.1.5 variant were recommended for the 2023–2024 vaccination season, reflecting the continued evolution of the virus and the need for variant-specific immune responses [32,33]. Real-world effectiveness studies of these variant-adapted vaccines have demonstrated their superior performance against circulating strains compared with the original monovalent boosters. A large-scale study showed that bivalent boosters provided 30% additional protection against symptomatic infections with XBB/XBB.1.5 variants compared with monovalent boosters [34]. Similarly, the updated 2023–2024 vaccines showed enhanced neutralizing activity against emerging variants, including JN.1, which became dominant in late 2023 [35,36].

One of the key findings of our study was that equivalent neutralizing antibody responses did not translate into equivalent clinical protection. In addition, systemic neutralizing antibody titers may not adequately capture mucosal immunity, which is essential for blocking viral entry at the upper respiratory tract. Clinical studies have shown that intramuscular mRNA vaccination induces only limited mucosal IgA responses [37]. This suggests that serum antibody levels alone may not reliably predict protection against breakthrough infection. Beyond immune escape by viral variants, other immunological factors may also limit the reliability of neutralizing antibodies as correlates of protection. In particular, although the majority of participants were classified as high responders (≥90% inhibition), their risk of breakthrough infection was not reduced compared with non-high responders, indicating that even very high neutralizing activity did not confer additional protection during the Omicron surge. A large Los Angeles cohort study of 859 participants found no association between receptor-binding domain antibody levels and breakthrough infection risk, with breakthrough rates of 19–23% regardless of antibody levels [38]. Similarly, Japanese HCWs showed no significant differences in pre-infection neutralizing antibody levels between breakthrough cases and controls among third-dose recipients [39]. This disconnect likely reflects the multifaceted nature of immune protection, which extends beyond circulating neutralizing antibodies to include cellular immunity, mucosal responses, antibody quality, and the kinetics of immune activation [40]. Our observation that the A-P-P regimen showed the highest breakthrough rates despite robust serological responses suggests fundamental differences in immune quality, rather than quantity. Vector-based vaccines may induce different patterns of memory B cell formation and T cell responses compared with mRNA platforms, potentially affecting the durability and breadth of protection [41]. The timing of the immune response development may also contribute to this paradox. While neutralizing antibodies were measured 1 month post-booster vaccination, the kinetics of immune activation and the establishment of protective immunity may differ between vaccine platforms. Studies have shown that breakthrough infections with Omicron variants primarily evoke immune responses targeting conserved epitopes rather than variant-specific sites, suggesting that initial immune imprinting from different vaccine platforms may influence subsequent protection patterns [42].

This study has some limitations. First, the number of participants and baseline characteristics, such as age, sex, and follow-up duration, were significantly different. This may have introduced bias in the comparison of breakthrough infection risks. Participation was voluntary, with 64.4% of eligible HCWs enrolled, so selection bias cannot be excluded. In addition, vaccine regimens were determined by national policy and occupational role, creating potential confounding between vaccine type and exposure risk. For example, an infection-free survival analysis using the booster date as the start of follow-up suggested superior infection-free survival in the P-P-P group (Figure 4A); however, this effect may have been overestimated because this group received boosters before the Omicron surge. To address this, we performed an additional analysis using a common start date after the completion of the booster vaccination (Figure 4B). Although the overall group differences were attenuated, the A-P-P group showed the lowest infection-free survival, and the relative advantage of the P-P-P group remained. In this context, the apparently superior performance of P-P-P may have been underestimated in the common start date analysis because other groups were assessed at shorter intervals after boosting and likely had higher baseline antibody levels. Although the A-P-P group had a higher median age, this difference is unlikely to reflect a clinically meaningful impairment of immune function, as vaccine responses are generally preserved until later adulthood. Age and sex were adjusted for in our Cox model, and analysis by clinical versus non-clinical staff did not reveal significant differences (Supplementary Figure S4). Nevertheless, as community exposure was not captured and the A-P-P group was small, the apparent excess risk in this group should be interpreted with caution. Second, the single-center design limits the generalizability of our findings. Third, our dataset did not capture detailed information on the clinical severity of breakthrough infections, so the potential role of T cell responses in mitigating disease severity could not be evaluated. However, conducting the study in a homogeneous HCW population provided strengths in exposure assessment and case confirmation, as surveillance and infection control measures were consistently applied. The differences among the vaccine groups should also be interpreted in the context of rapidly evolving national vaccine policies rather than methodological shortcomings. Although not equivalent to randomized controlled trial conditions, these data represent a natural experiment that reflects real-world vaccination strategies during the dynamic transition period in Republic of Korea. In addition, neutralization was assessed using the cPass sVNT, which employs the ancestral Wuhan strain RBD. While this assay correlates well with PRNT, it does not capture non-RBD epitopes and may not fully reflect protection against Omicron subvariants carrying >30 spike mutations [3,43]. Thus, the correlation between post-booster sVNT levels and breakthrough infection risk should be interpreted with caution.

Despite these limitations, this study has several strengths. This prospective cohort captured a natural experiment during the Omicron surge in Republic of Korea, reflecting actual national vaccine allocation policies, direct comparison of four regimens in a homogeneous HCW population, with standardized exposure assessment and systematic surveillance. Although residual confounding by demographic factors and follow-up differences could not be fully controlled, randomized controlled trials under idealized conditions cannot fully predict vaccine performance in urgent, rapidly changing public health contexts. In contrast, real-world analyses such as ours provide valuable evidence of how vaccines perform under actual implementation conditions, complementing controlled trial data. The observed “rescue effect” of mRNA boosters, restoring neutralizing antibody levels irrespective of the primary regimen, supports flexible strategies adopted during the ChAdOx1 phase-out. Moreover, a systematic collection of immunogenicity and breakthrough outcomes offers baseline data that may inform preparedness for future pandemics. Real-world observations, including unexpected findings encountered during policy transitions, can provide important academic insights for guiding responses to emerging infectious diseases. Further studies should evaluate the durability of heterologous booster responses, optimal timing, and dosing intervals, and potential benefits of variant-specific boosters in mixed-platform regimens.

## 5. Conclusions

Heterologous mRNA boosters restored neutralizing antibody levels across all primary regimens. However, regimens including adenovirus-vectored vaccines, particularly the A-P-P combination, showed higher breakthrough infection rates during the BA.1/BA.2 Omicron surge, suggesting that neutralizing titers alone may not fully predict protection. Vaccine development should therefore aim not only to maximize neutralizing antibodies but also to consider broader and potentially more durable immune responses, such as T cell-mediated immunity suggested in previous studies. Public health policy should likewise emphasize prospective monitoring of real-world effectiveness during booster rollouts, as immunological data alone may be misleading.

## Figures and Tables

**Table 1:** Baseline characteristics of study participants according to booster vaccine group.

Characteristics	Total (*n* = 318)	P-P-P (*n* = 71)	A-A-P (*n* = 205)	A-P-P (*n* = 19)	M-M-M (*n* = 23)	*p*-Value
Age, years, median ± IQR	32 (27–44)	28 (26–37)	35 (27–49)	41 (34–44)	25 (23–31)	0.000
Sex: Female, n (%)	238 (74.8)	42 (59.2)	159 (77.6)	15 (78.9)	22 (95.7)	0.001
Occupation: Clinical, n (%)	234 (73.6)	59 (83.1)	152 (74.1)	5 (26.3)	18 (78.3)	0.000
Pre-booster infection, n (%)	7 (2.2)	1 (1.4)	3 (1.5)	2 (10.5)	1 (4.3)	0.068
Follow-up duration, days, median (IQR)	154 (110–220)	249 (147–277)	144 (113–220)	84 (81–94)	95 (80–190)	0.000

Clinical = patient-facing occupation (such as doctors, nurses, and direct patient care). Non-clinical = non-patient-facing occupation (such as administration, cleaning, and support staff).

**Table 2:** Neutralizing antibody inhibition (%) and seropositive rates before and after booster vaccination.

Outcome	P-P-P (*n* = 71)	A-A-P (*n* = 205)	A-P-P (*n* = 19)	M-M-M (*n* = 23)	*p*-Value
Before booster					
Inhibition (%), median (IQR)	82 (68–91)	40 (25–62)	80 (49–87)	93 (91–94)	0.000
Seropositive rate n (%)	70 (98.6)	132 (64.4)	17 (89.5)	23 (100.0)	0.000
1-month post-booster					
Inhibition %, median (IQR)	97 (97–97)	97 (97–97)	97 (97–97)	97 (97–97)	0.002
Δ Absolute change (V2–V1), median (IQR)	15 (5–30)	55 (34–71)	18 (10–47)	3 (2–6)	0.000
Seropositive rate, n (%)	71 (100.0)	204 (99.5)	19 (100.0)	23 (100.0)	0.907
High responder, n (%)	69 (97.2)	199 (97.1)	19 (100.0)	23 (100.0)	0.741
Breakthrough infection no event (%)	31 (43.7)	115 (56.1)	16 (84.2)	14 (60.9)	0.0137
Inhibition % (median) at post-booster, by infection status	97.2 vs. 97.2	96.9 vs. 97.0	97.0 vs. 96.9	96.7 vs. 96.6	NS

*p*-values were obtained using the Kruskal–Wallis test for continuous variables and the chi-square test for categorical variables. Post hoc pairwise comparisons were conducted, and significant differences are described in the Section 3. Seropositive is defined as inhibition ≥30%; High responder is defined as inhibition ≥90%: NS, no significant difference between infected and non-infected patients (Mann–Whitney U test). IQR, interquartile range.

## Data Availability

The data presented in this study are available on request from the corresponding author. The data are not publicly available due to institutional privacy and ethical restrictions.

## Supplementary Materials

The following supporting information can be downloaded at: https://www.mdpi.com/article/10.3390/microorganisms13102362/s1, Figure S1. Schoenfeld residual plots for booster-date Cox model. Scaled Schoenfeld residuals for each regimen covariate: (A) A-A-P, (B) A-P-P, (C) M-M-M. Figure S2. Schoenfeld residual plots for common-date Cox model. Scaled Schoenfeld residuals for each regimen covariate: (A) A-A-P, (B) A-P-P, (C) M-M-M. Figure S3. log(−log) infection-free survival plots for booster regimens. (A) Booster-date start, (B) Common-date start. Figure S4: Kaplan–Meier infection-free survival curves for breakthrough infection stratified by occupational category (clinical vs. non-clinical). There was no significant difference between the two groups (log-rank *p* = 0.43).

## Abbreviations

The following abbreviations are used in this manuscript:

COVID-19	Coronavirus disease 2019
SARS-CoV-2	Severe acute respiratory syndrome coronavirus-2
HR	Hazard ratio
CI	Confidence interval
HCWs	Health care workers
IQR	Interquartile range
KDCA	Korea Disease Control and Prevention Agency
