# Supplementary material for: Immunogenicity and Breakthrough Outcomes of mRNA Booster Strategies Among Healthcare Workers During the BA.1/BA.2 Omicron Surge
Source: Microorganisms. 2025 Oct 14;13(10):2362. doi: 10.3390/microorganisms13102362 (PMC12565756; doi:10.3390/microorganisms13102362)
Supplement: Supplementary file 1 [file microorganisms-13-02362-s001.zip › microorganisms-3859070-supplementary.pdf]

Supplementary Figures

Figure S1. Schoenfeld residual plots for booster-date Cox model.

Scaled Schoenfeld residuals for each regimen covariate: (A) A-A-P, (B) A-P-P, (C) M-M-M.

Figure S2. Schoenfeld residual plots for common-date Cox model.

Scaled Schoenfeld residuals for each regimen covariate: (A) A-A-P, (B) A-P-P, (C) M-M-M.

Figure S3. log(–log) survival plots for booster regimens.

(A) Booster-date start, (B) Common-date start.

Figure S4. Kaplan–Meier infection-free survival curves stratified by occupational group.

Infection-free survival was compared across occupational categories.

Figure S1

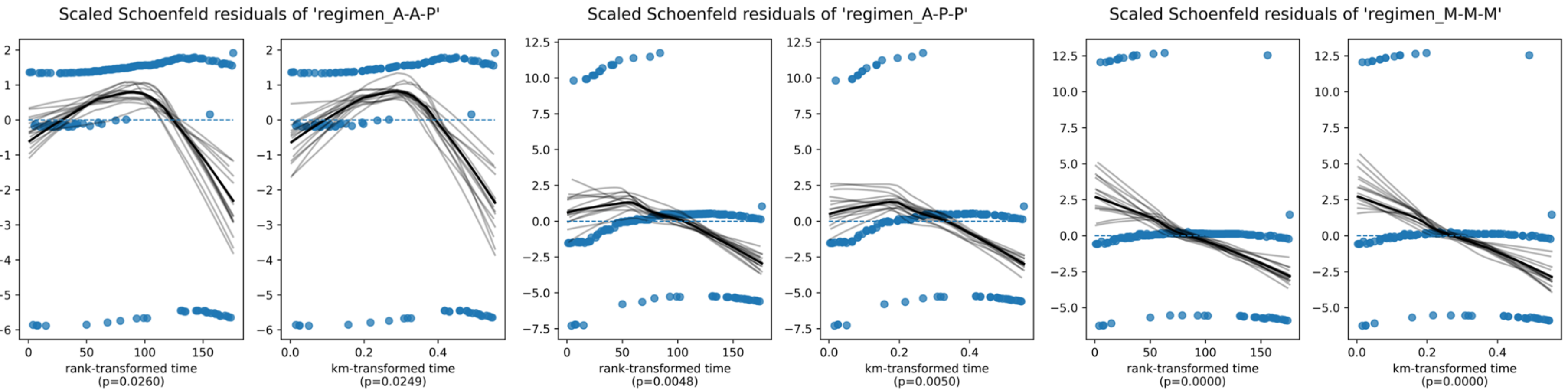

Figure S2

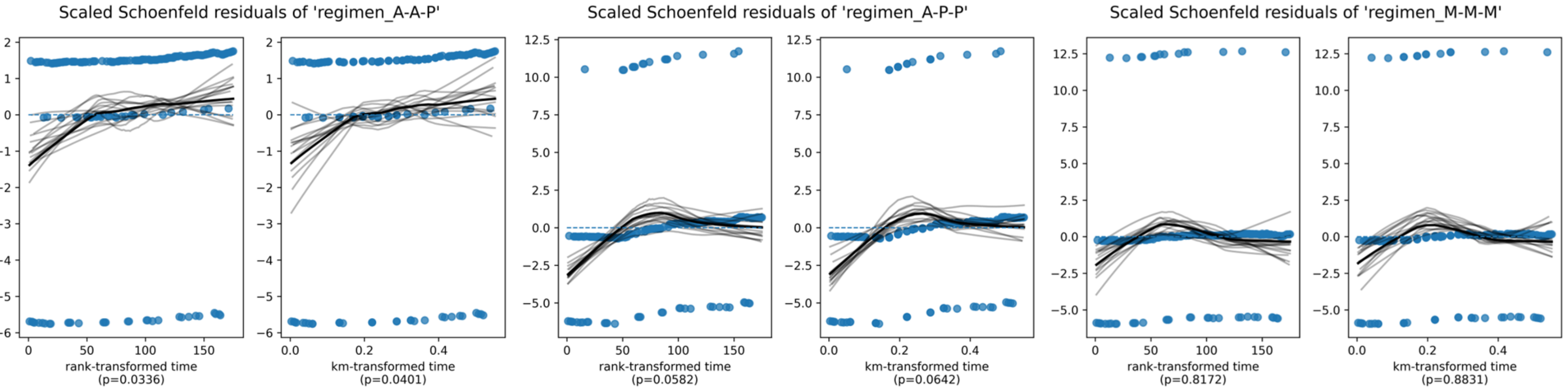

Figure S3

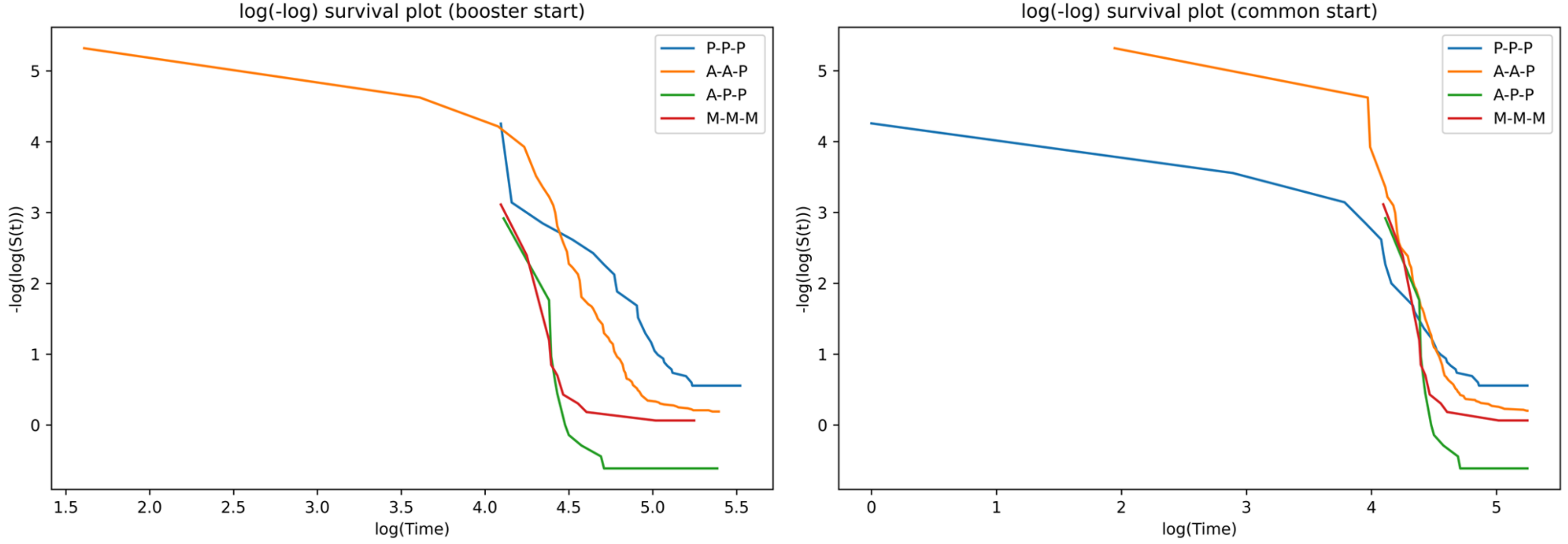
log(-log) survival plot (booster start)
log(-log) survival plot (common start)
P-P-P
A-A-P
A-P-P
M-M-M
-log(log(S(t)))
log(Time)
1.5
2.0
2.5
3.0
3.5
4.0
4.5
5.0
5.5
0
1
2
3
4
5

Figure S4

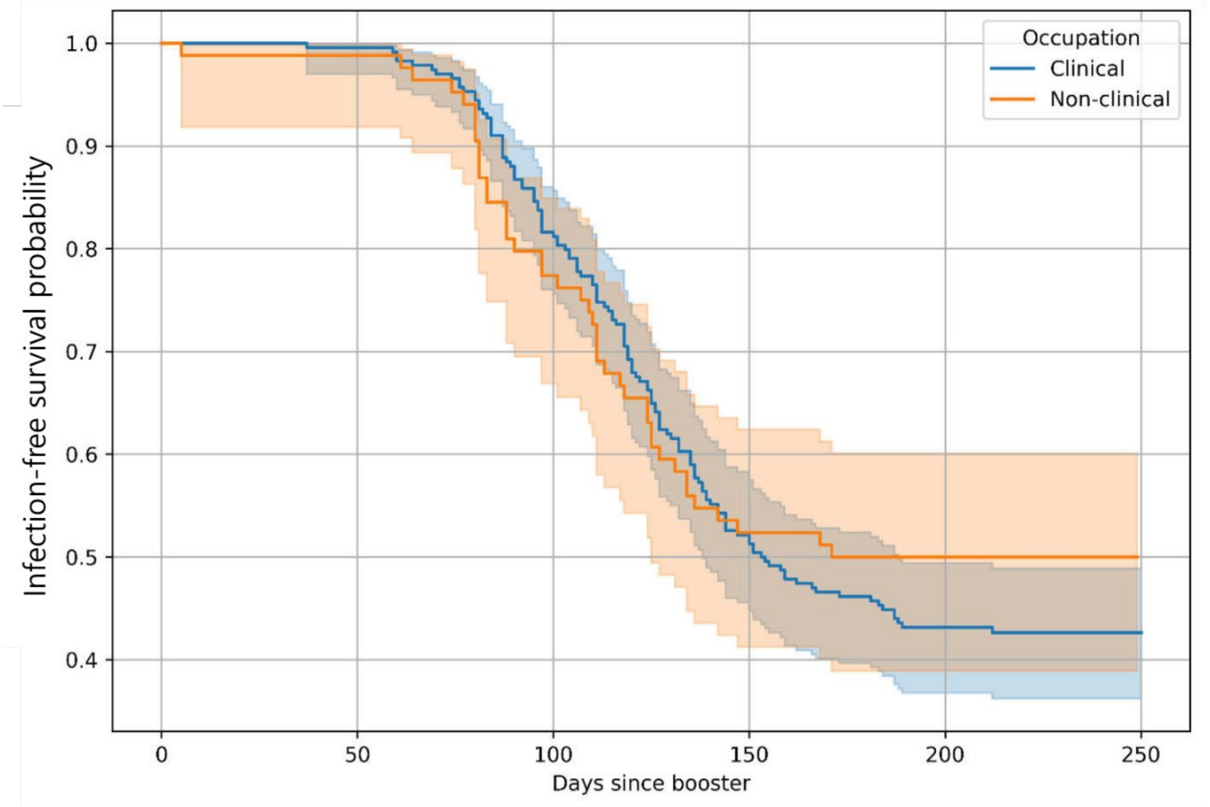
Occupation
Clinical
Non-clinical
Infection-free survival probability
1.0
0.9
0.8
0.7
0.6
0.5
0.4
0
50
100
150
200
250
Days since booster
